# Detecting Retroviral Sequences in Chronic Fatigue Syndrome

**DOI:** 10.3390/v2112404

**Published:** 2010-11-03

**Authors:** Ila R. Singh

**Affiliations:** Department of Pathology, University of Utah, Salt Lake City, UT 84112, USA; E-Mail: ila.singh@path.utah.edu; Tel.: +1 801-213-3737

**Keywords:** polytropic and modified polytropic viruses, XMRV, PMV, M-PMV

## Abstract

XMRV or xenotropic murine leukemia virus-related retrovirus, a recently discovered retrovirus, has been linked to both prostate cancer and chronic fatigue syndrome (CFS). Recently, the teams of Drs. Shyh-Ching Lo and Harvey Alter discovered the presence of sequences closely related to XMRV in the blood of 86.5% of patients with CFS [1]. These findings are important because since the initial discovery of XMRV in CFS, several studies have failed to find XMRV in specimens collected from CFS patients. While the current study also did not find XMRV in CFS, Lo *et al.* did detect sequences that belong to polytropic mouse endogenous retroviruses (PMV), which share considerable similarity with XMRV. Criteria for future studies that will help bring greater clarity to the issue of retroviral sequences in CFS are proposed below.

XMRV has been linked to both prostate cancer [2–5] and CFS [6]. Recently, Lo *et al* discovered the presence of sequences closely related to XMRV in the blood of 86.5% of patients with CFS. These findings are important because since the initial discovery of XMRV in CFS, there have been several studies that failed to find XMRV in CFS, in the U.S. [7], in Europe [8–10] and in China [11]. While the current study also did not find XMRV in CFS, Lo *et al.* did detect sequences that belong to polytropic mouse endogenous retroviruses (PMV).

While each of these viruses, xenotropic, polytropic and modified-polytropic viruses (M-PMV), has been placed in distinct categories within the larger subgroup of murine leukemia viruses, it is important to note that they share considerable similarity with each other and utilize alleles of the same receptor for viral entry. The PMV and M-PMV share over 95% sequence identity, and the xenotropic viruses share over 90% identity with the PMV (derived from sequences within reference [12] and Genbank; [13,14]). Compared to this, an average pair of random isolates of HIV-1 in the U.S. are ∼85–95% similar, depending on the region of the viral genome used for comparison [15,16]. Thus it is conceivable that PMV, M-PMV and XMRV may cause the same disease(s), if they cause disease at all.

It seems unlikely that the polytropic sequences detected by Lo *et al.* are due to contamination with mouse DNA. The PCR used to detect mouse mitochondrial DNA was reported to have a hundred-fold higher sensitivity than the PCR used to detect the MLV *gag* gene. Also, over 300 negative controls were consistently negative. Furthermore, the positivity rate among healthy volunteers was only 6.8%, considerably smaller than the 86.5% seen in patients and improbably low if contamination was really an issue. However, it is important to note that the CFS patients were from Massachusetts while the controls were from Maryland. Regional differences in sequences among prevailing viruses could easily result in one set being detected well by PCR, and another not.

Proving that xenotropic or polytropic viruses are present in chronic fatigue syndrome would be the first step towards determining if these viruses actually *cause* disease. This is an important question because a large fraction of the world population, estimated at 0.4 to 1%, is affected by CFS [17]. Faced with a chronic, severely debilitating illness and the lack of effective and approved diagnostic or therapeutic options, some patients have begun treatment with antiretroviral agents, based on effects of these drugs on XMRV replication *in vitro* [18–21]. Whether this is a wise course of action to take—given the inconsistencies in available data—can be debated. But what cannot be debated is that there is a real need for thoughtfully designed and carefully executed studies that examine this issue more thoroughly.

At this point, it might be good to evaluate parameters essential to resolve whether XMRV and other related viruses are truly present in specimens taken from CFS patients. A good study will need a sufficiently large set of patients that fulfill well-recognized criteria for CFS, such as those outlined by the 1994 case definition of the CDC [22], as well as the criteria defined by the Canadian consensus document on myalgic encephalitis/CFS [23]. From this set of patients, those taking antiretroviral drugs should be excluded, as a negative result could simply mean that viral titers have decreased to an undetectable range by the drugs. It is also essential that the control population is large, and from the same geographical area as the patients. In both the current study and the original CFS-XMRV study (by Lombardi *et al.* [6]), the patients and controls were from geographically distinct locations in the U.S. Furthermore, it is important that patient samples are collected and treated the same way as controls. Most patient samples in the Lo *et al.* study [1] came from material frozen ∼15 years ago, while the controls were all recently acquired. A smaller subset (21%) of patient samples were collected more recently, and results from their analysis corroborated those from older, stored samples. However, this was a very small set, consisting of only eight samples. In a study that found a contaminating rabbit betaretrovirus in rheumatoid arthritis patients but not in controls [24], the lead author, Robin Weiss, contends that patient samples may have been handled much more than controls, allowing for additional chances for contamination [25]. Blinding the investigator to the identity of patient samples and controls would add an additional measure of confidence to the results. Another important factor in designing future studies would be making sure that there are good controls for each step of the analysis. For example, negative controls for PCR, which usually consist of water, should also go through the same nucleic acid extraction process as all human samples used in the study. Thus, contamination of samples occurring prior to amplification is likely to be detected as a positive PCR from a ‘water’ control. Furthermore, the number of negative controls per plate should at least equal if not exceed the prevalence of the virus in the control population. It is also important to characterize methods of detection in more detail than they have been so far. The present study describes their nested PCR assays as being able to detect a single copy of retroviral DNA. However, we are not told how reproducible this limit of detection is, either within a single run or in successive runs. We also do not know how specific this PCR is, as in what other retroviral genomes might result in PCR products of similar size, though the authors do sequence each product of appropriate size, thereby demonstrating that only polytropic mouse virus sequences were detected. It is important to recognize that PCR-based assays, the assays used in this study, can only detect nucleic acid —which may represent replicating virus, or partial retroviral sequences or simply nucleic acid contaminants. Other assays, such as isolating replicating virus from plasma or detecting specific antibodies in serum will be important corroborating tests to prove the presence of an infectious virus in a set of patients. Finally, an ideal study would use at least one method that has resulted in detection of XMRV in a previous study, preferably using a set of samples that were analyzed in that same previous study and are ‘known’ positives or negatives. In order to avoid any possibility of contamination, it is important that these shared samples not go to any research lab for patient deidentification or aliquoting, but instead go from the phlebotomy lab directly to the researchers who will do the testing. A study that incorporates these criteria is likely to resolve more clearly whether xenotropic or polytropic viruses are present in chronic fatigue syndrome, and move us a step closer to determining if these viruses cause disease.
